# 

**Published:** 1889-12

**Authors:** 


### TABLE OF CONTENTS.

ORIGINAL COMMUNICATIONS. PAGE

A Study of Electricity, with the View of comprehending its Application in Dentistry. By Howard E. Roberts, D.D.S., Philadelphia . . . . . . 703

Pyorrhœa Alveolaris. By George S. Allan, D.D.S., New York City . 712

Light to the Gentiles. By W. S. Elliott, M.D., D.D.S., M.D.S. . . . . 726

Sensation. By George W. Whitefield, M.D., D.D.S., Chicago . . . . 731

REPORTS OF SOCIETY MEETINGS.

Pennsylvania State Dental Society . . . . . . . . . . . . . . . . . . 734

New Jersey State Dental Society . . . . . . . . . . . . . . . . . . 739

EDITORIAL.

Our Position . . . . . . . . . . . . . . . . . . . . . . . . . 752

A New Use for the Phonograph . . . . . . . . . . . . . . . . . 754

Our Closing Reply on Trade Journalism . . . . . . . . . . . . . 756

FOREIGN CORRESPONDENCE . . . . . . . . . . . . . . . . . 758

DOMESTIC CORRESPONDENCE . . . . . . . . . . . . . . . . 761

CURRENT NEWS.

Chicago and Vicinity . . . . . . . . . . . . . . . . . . . . . . 764

Items . . . . . . . . . . . . . . . . . . . . . . . . . . . . . 765

National Association of Dental Faculties . . . . . . . . . . . . . 766

